# A Mixed Method Systematic Review Into the Impact of ED Treatment in Autistic People and Those With High Autistic Traits

**DOI:** 10.1002/eat.24311

**Published:** 2024-11-14

**Authors:** Emy Nimbley, Helen Sharpe, Ellen Maloney, Karri Gillespie‐Smith, Kate Tchanturia, Fiona Duffy

**Affiliations:** ^1^ Department of Psychology, School of Health in Social Science University of Edinburgh Edinburgh UK; ^2^ Department of Philosophy, School of Philosophy, Psychology and Language University of Edinburgh Edinburgh UK; ^3^ Department of Psychological Medicine Institute of Psychiatry, Psychology and Neuroscience, Kings College London London UK; ^4^ Eating Disorder Development Team NHS Lothian Child and Adolescent Mental Health Services, Royal Edinburgh Hospital Edinburgh UK

**Keywords:** autism, eating disorders, mixed‐methods, treatment

## Abstract

**Objective:**

Our understanding of the impact of eating disorders (ED) treatment in Autistic people remains elusive. Research has begun to explore ED treatment outcomes and experiences in this population, however current understandings are poorly integrated. The current review therefore sought to explore the impact of ED treatment on Autistic people and those with higher Autistic traits.

**Method:**

A convergent, segregated approach was used, independently evaluating quantitative then qualitative studies before integrating findings into a coherent narrative synthesis.

**Results:**

Autistic people and people with higher Autistic traits report poorer experiences of treatment and may be at increased risk of inpatient admission and prolonged inpatient treatment, possibly explained by difficulties with treatment timeframes and a lack of autism‐informed support. Both groups reported similar improvements in ED symptoms and BMI. Higher rates of psychosocial difficulties pre‐and post‐treatment were reported in those with higher Autistic traits, and emotion‐focused interventions were felt to be particularly relevant to Autistic presentations of EDs. Concerns were reported as to how well aligned group‐based programs and cognitive‐based interventions are for Autistic individuals and those reporting higher Autistic traits.

**Discussion:**

Future research in diagnosed autism samples is urgently needed to develop a more robust understanding of Autistic outcomes and experiences. Review findings demonstrate the need for increased understanding of ED presentations and the possible need for treatment adaptations, for Autistic people or those with higher Autistic traits.

**Summary**

- This review evaluates the current evidence looking at the impact of ED treatment for Autistic people and those with higher Autistic traits, drawing on both measurable outcomes (quantitative data) and lived experience narratives (qualitative data).
- It is hoped that we can provide novel insights into the impact of ED treatment on Autistic people and those with higher Autistic traits and begin to translate this into improving support and wellbeing.

## 1 Introduction

Eating and feeding difficulties, such as food selectivity, are highly common in Autistic children, and adolescents (Sharp et al. 2013; Bandini et al. 2017). Concerningly, there is increasing evidence suggesting that not only do such eating and feeding behaviors persist across development (e.g., Baraskewich et al. 2021) but that Autistic individuals may be at a heightened risk of developing an eating disorder (ED); Lai et al. 2019; Bourne et al. 2022). Self‐identifying Autistic individuals are twice as likely to experience EDs compared to neurotypical peers (Sedgewick, Leppanen, and Tchanturia 2021), and prevalence estimates of Autistic traits in anorexia nervosa (AN) are between 20% and 40% (Mandy and Tchanturia 2015; Westwood and Tchanturia 2017; Spek et al. 2019; Huke et al. 2013). Elevated Autistic traits have similarly been reported in bulimia nervosa (BN), binge eating disorder (BED) and avoidant and restrictive food intake disorder (ARFID) (Gesi et al. 2020; Koomar et al. 2021).

Some have argued that the high prevalence of Autistic traits in ED populations may be a behavioral consequence of having an ED via starvation or social withdrawal (e.g., Treasure 2013). However, several lines of research suggest otherwise. Firstly, there is consistent evidence to support that BMI is not associated with Autistic traits in AN samples (Bentz et al. 2017; Tchanturia et al. 2019; Nazar et al. 2018; Sedgewick et al. 2019; Susanin et al. 2022; Pruccoli et al. 2021), and higher Autistic traits have been reported in non‐restrictive EDs, such as BED (Gesi et al. 2020). Several studies also offer longitudinal evidence to suggest that Autistic traits in childhood both precede and predict the development of disordered eating in adolescence (Solmi et al. 2021; Leno et al. 2022; Karjalainen et al. 2019), with qualitative evidence supporting that Autistic traits are present earlier in development (e.g., Brede et al. 2020). Collectively, these studies suggest that the elevated rates of Autistic traits reported in studies are not solely attributable to secondary features of an ED.

The degree, to which such studies reflect a true overlap between autism and EDs, however, remains to be determined. While some studies have reported elevated Autistic traits in those who have recovered from AN (Nazar et al. 2018; Bentz et al. 2017; Sedgewick et al. 2019), others have reported a reduction in Autistic characteristics following treatment and weight restoration (Nuyttens et al. 2024; Susanin et al. 2022). Importantly, these studies also report a significant percentage of participants scoring above screening thresholds for autism following treatment (e.g., Nuyttens et al. 2024). A recent paper found similar clinical characteristics, such as ED symptoms, sensory processing, social differences, and mental health difficulties, in both diagnosed samples and those with elevated Autistic traits (Brede et al. 2024). Thus, these findings suggest that elevated Autistic characteristics reported across studies likely reflect a complex combination of both traits and states (Kerr‐Gaffney et al. 2021; Saure et al. 2020; Nuyttens et al. 2024). Importantly however, considering the reported similarities in presentation (Brede et al. 2024), there is a common need for greater understanding of ED treatment outcomes and experiences in both Autistic people and those with higher Autistic traits.

Recent research has started to focus on identifying possible mechanisms underlying the elevated rates of autism and Autistic traits observed in ED samples. Given emerging longitudinal evidence (Solmi et al. 2021; Leno et al. 2022), it could be that autism or higher Autistic traits constitute a specific risk factor for developing an ED, or that both conditions share causal pathways (see Adams et al. 2024 for a more detailed summary of this debate). Several shared mechanisms have been reported, including social cognition (Zucker et al. 2007; Kerr‐Gaffney et al. 2020; Leppanen et al. 2018; Sedgewick et al. 2019), broader social functioning (Kerr‐Gaffney et al. 2021) and cognitive traits (Westwood et al. 2016; Lang et al. 2014). A recent model of autism‐specific cognitive, social, emotional, and sensory differences has been proposed (Brede et al. 2020), however many of these mechanisms have not yet been tested empirically. Identifying common or condition‐specific mechanisms has important implications on enhancing our understandings of similarities and differences in ED treatment experiences and on identifying appropriate treatment targets. ED clinicians report a lack of experience and/or awareness of autism‐specific needs such as communication and sensory differences (Li et al. 2022a; Li et al. 2022b) and a lack of confidence in delivering treatments to Autistic individuals (Kinnaird, Norton, and Tchanturia 2017). Gaining a comprehensive understanding of the experience and effects of existing ED treatments for Autistic individuals and those with higher Autistic traits is a key first step in being able to effectively support this population.

Several reviews have previously been conducted to explore the overlap between autism and EDs (Huke et al. 2013; Westwood and Tchanturia 2017; Bolti and Sapuppo 2021), with a recent review exploring the impact of Autistic traits on broad ED outcomes (Li et al. 2022a). The current review will extend these findings in several ways. Firstly, it will adopt a broad consideration of outcomes impacted by ED treatment and conceptualized in recovery, focusing not only on ED‐specific outcomes such as cognitions, behaviors, and BMI, but also on broader markers of recovery, such as psychological wellbeing and social functioning (Wetzler et al. 2020; Richmond et al. 2020; de Vos et al. 2017). Second, the current review will adopt a mixed‐method approach in order to contextualize clinically measurable outcomes within lived/living experience perspectives and to explore if these complement or contradict each other. Such mixed methods inquiry is being increasingly adopted in health and psychological research (Regnault et al. 2017; Creamer and Repping 2020), generating findings that are rooted in patient‐identified priorities. Drawing on both quantitative and qualitative evidence, the current review will seek to understand the subjective and objective impact of ED treatment on Autistic individuals and those with higher Autistic traits. Specifically, the review will address the following research questions:

1. What are ED treatment outcomes for Autistic individuals/those with higher Autistic traits?
2. What are the experiences of ED treatment outcomes for Autistic individuals/those with higher Autistic traits, and what can they tell us about the likely impact of ED treatment on this population?

Please note, the following manuscript will use identity‐first language (e.g. Autistic person, non‐Autistic person) as opposed to person‐first language (e.g., person with autism). This is in line with well‐evidence preferences of the Autistic community (e.g. Kenny et al. 2016; Bury et al. 2023; Bottema‐Beutal et al. 2021; Monk et al. 2022) and with neurodiversity and Autistic rights movements who position autism as a complex interplay between identity and difference (Leadbitter et al. 2021).

## 2 Methods

The current mixed‐methods review was conducted in line with Preferred Reporting Items for Systematic Reviews and Meta‐Analyses (PRISMA) guidelines (Page et al. 2021) including both qualitative and quantitative studies. A convergent, segregated design was chosen, whereby both lines of enquiry are analyzed independently (segregated) before converged into an integrated synthesis of evidence (convergent). This approach involves the independent synthesis of quantitative and qualitative data, before the integration of evidence from each synthesis (Pearson et al. 2015; Hong et al. 2017; Sandelowski et al. 2010; Stern et al. 2020). This approach was chosen due to the focus of the review being on different aspects of the same phenomena of interest; specifically, looking at clinically measurable outcomes or experiences of ED treatments, and if or how these align with lived/living experiences of ED treatment. Through this strategy, the review aimed to see whether qualitative and quantitative data complements, conflicts, or contradicts each other (Stern et al. 2020; Pearson et al. 2015). The review aimed to summarize the quantitative studies and reported outcomes for Autistic people and those with higher Autistic traits with an ED, before summarizing the qualitative studies to explore whether lived experiences of ED treatment complement or contradict reported findings. Accordingly, studies will be summarized separately (study characteristics, key results) before being integrated into a coherent narrative synthesis of mixed‐method findings. The protocol for the review was registered on PROSPERO (CRD42023396368).

### 2.1 Eligibility Criteria

Quantitative studies were eligible for inclusion if they reported on primary or secondary data on ED and/or broader outcomes and experiences (e.g., cognitive outcomes) directly associated with all levels of ED services (e.g., inpatient/outpatient/day patient services) or treatments (e.g., cognitive behavioral therapy, emotion‐focused therapies, occupational therapies) in Autistic individuals or those with higher Autistic traits. Qualitative studies were included if they explored living/lived experiences of ED treatment and services for Autistic individuals. Both clinical and community samples were included across studies, seeking to encompass a broad range of ED services (e.g., inpatient, outpatient, day services). There was no exclusion made on dates, and both published and gray literature were considered. Non‐empirical publications (e.g., reviews, meta‐analyses, book chapters, editorials, conference proceedings, non‐English language, case studies) were not included. Full text was required to be in English.

Studies were eligible for the review if they included both clinically diagnosed Autistic samples and those with higher Autistic traits, identified using a standardized measure. The reliance of research on Autistic traits as an indicator of autism has raised concerns regarding the validity of findings and a possible artefactual association between the two presentations (see Adams et al. 2024). Recent evidence suggests, however, that both diagnosed and those who have higher Autistic traits show similar patterns of disordered eating behaviors and autism‐specific behaviors compared to Autistic only and restrictive ED only samples (Brede et al. 2024). Studies with both formal and self‐identified Autistic samples were also included in this review. This is due to the significant barriers to referrals for autism diagnostic assessments (Overton, Marsa‐Sambola, Martin & Cavenagh, 2023) and reported waiting times of up to 8 years (Babb et al. 2021). Additionally, it is reported that those who self‐identify as Autistic, score similarly on autism and quality of life scales compared to those with clinical diagnoses of autism, and that those without a clinical diagnosis are often the marginalized minorities within the neurominority (McDonald 2020). This inclusive approach also mirrors recent thinking in the autism field, moving away from a rigid categorical approach to autism (Fletcher‐Watson 2022), reflecting a pragmatic approach commonly adopted by clinical services to quickly make support more accessible for a wider range of individuals (Brede et al. 2024).

### 2.2 Information Sources And Search Strategies

Following an initial scoping search, four databases were searched: PsychINFO (searched through OVID interface), PubMed, Scopus and Web of Science. Unpublished theses and dissertations were searched for using ProQuest Dissertation and Theses. Databases were initially searched in September 2023 and were last searched in September 2024. Citation chaining was conducted on the reference list of screened papers. Search terms included autism OR ASD AND ED OR anorexia OR bulimia OR binge eating OR ARFID AND treatment OR services AND outcomes OR experiences OR perspectives.

### 2.3 Study Selection And Data Collection

Two reviewers (EN and FD) independently screened titles and abstracts, before screening all potentially eligible full texts against inclusion criteria. Both reviewers screened all texts, and any disagreements would be resolved by mutual agreement. Any full texts that did not meet inclusion criteria were excluded. Data was extracted based on the JBI Mixed Methods Data Extraction Form, including information regarding study type, study aims, methodology, total number of participants, participant characteristics, type of ED, autism status (diagnosis or traits), phenomena of interest, setting/context, results/themes (with quotations) and study conclusions.

### 2.4 Critical Appraisal

Two reviewers (EN and FD) conducted a critical appraisal on individual studies using the Mixed Methods Appraisal Tool (MMAT; Hong et al. 2018). The MMAT tool is an effective tool for assessing the quality of studies in a mixed‐method review, with five questions for each study design. Papers were rated independently, and inter‐rater reliability (Cohen's kappa) was calculated to be 0.82, indicating moderate agreement (McHugh 2012). Any differences at this stage were minor (e.g., discrepancies between yes and can't tell, or no and can't tell) and were resolved by mutual agreement.

### 2.5 Effect Measures

Where possible, the results section will report effect sizes (see Tables 2 and 3). Due to the heterogeneity of included studies the results section will draw on a range of effect sizes, including partial eta squared (small = 0.01, medium = 0.06, large = 0.14), correlation coefficients (*r*; small = 0.10, medium = 0.30, large 0.50), odds ratios (OR; small = 1.1–1.5, medium = 1.5–3, large = 5.0 and above), Hedge's g (small = 0.20, medium = 0.50, large = 0.80), Cohen's d (small = 0.2, medium = 0.5, large = 0.8)and Nagelkerke's *R* ^2^ (small = 0.02 or less, medium = 0.2–0.4, large = 0.4 and above).

### 2.6 Synthesis Methods

In line with MMSR guidelines for convergent, segregated approaches, results synthesis will combine independent quantitative and qualitative findings into a coherent analysis. Results from quantitative findings will be narratively synthesized in each section first, followed by results from the qualitative findings. Quantitative data was extracted, narratively synthesized and organized into ED treatment outcome categories. Qualitative findings were distilled using a descriptive or content analytical approach and narratively synthesized into the same outcomes categories. This approach was chosen in order to explore whether reports of ED treatment experiences complement or contradict ED treatment outcomes, and to see if any aspects of each are not explored in the other (see Stern et al. 2020).

### 2.7 Researcher Reflexivity

EN extracted and synthesized the data. EN is a post‐doctoral researcher who has been conducting research and collaborating with the Autistic and ED community since her PhD exploring the overlap between autism and AN. EN is thus aware of the complexity of autism and EDs and how poorly this is understood, and is keenly motivated to enact evidence‐based change to improve the lives of Autistic people with an ED.

## 3 Results

**FIGURE 1:**
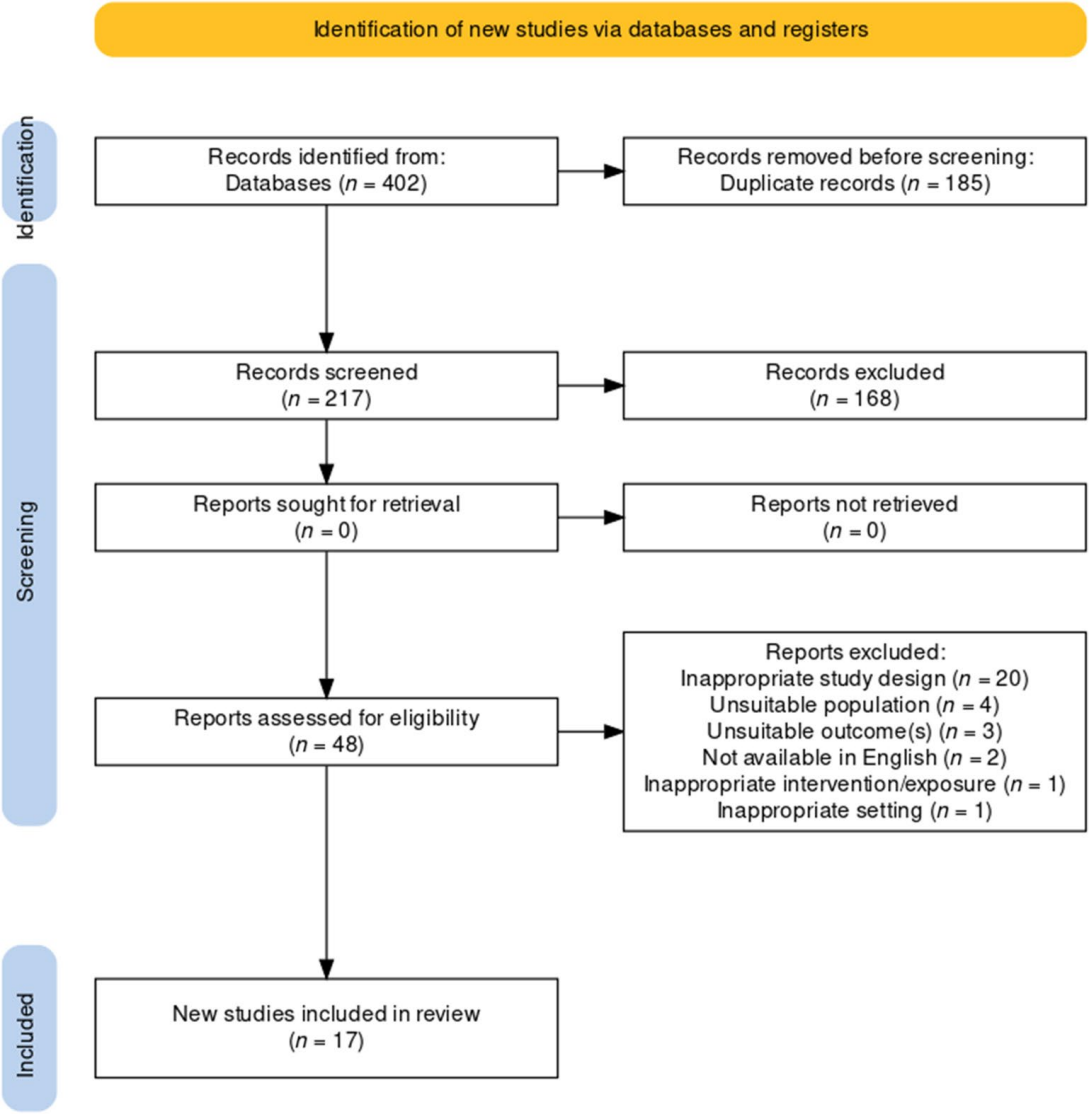
Prisma flow diagram for studies included in the review (*n* = 17).

A total of 17 studies were included in the review, comprising of 15 quantitative and two qualitative studies. Full details of the screening and inclusion of studies is displayed in Figure 1. References and abstracts of the two non‐English papers that were excluded can be found in Supporting Information A.

### 3.1 Study Characteristics

Study characteristics were extracted according to study type, design, control/comparison group, number of participants, female (%), ED (diagnosis), autism (diagnosis or traits measured by a standardized measure), ED services/treatments and outcomes, and can be found in Table 1. 14 studies looked at anorexia nervosa (AN) samples, and two looked at restrictive eating disorders (REDs) samples, which included AN, atypical AN and ARFID in Babb et al, (2021) and AN and atypical AN in Bentz et al (2022). One study looked at all EDs, including AN, bulimia nervosa (BN), binge eating disorder (BED), atypical AN, ARFID, purging disorder or unspecified feeding and ED. Six studies used Autistic samples that had received a diagnosis, two studies used a mixed sample of diagnosed and Autistic traits, while the remaining 9 studies looked at high Autistic traits using standardized measures (see Table 1).

**TABLE 1:** Study characteristics of included studies (*n* = 17), organized into studies that used Autistic traits (*n* = 9), autism diagnosis (*n* = 6), or both (*n* = 2) in their sample.^a^

Author	Methodology – methods	Control/comparison group	*N*	Female n (%)	Race – ethnicity *n* = (%)	Age range	ED	Autism measure	Services (treatment)	Outcome/phenomena of interest
Traits										
Adamson et al. (2018)	Quantitative – case series	AN only	CREST‐I = 66 (AN+ASD = 21) CREST‐G = 62 (AN+ASD = 21)	100%	—	18+	AN	Traits – AQ‐10	Inpatient (CREST)	Socio‐emotional outcomes, motivation for change
Dandil et al. (2020)	Quantitative – case series	Low autistic traits	Total AN = 99 (High autistic traits = 59)	59 (100%)	—	18+	AN	Traits – AQ‐10	Inpatient (CRT)	Cognitive outcomes
Giombini et al. (2022)	Quantitative – feasibility RCT	Non‐Autistic AN	Total AN = 80 (Autism = 18)	Total AN = 75 (93.8%)	White = 73 (91.4%), asian = 6 (7.5%, mixed white/black caribbean = 1 (1.3%)	10–18	AN	Traits – SCQ, SRS	Inpatient (CRT)	Cognitive outcomes, ED symptoms, depression, motivation for change, social communication, clinical outcomes
Huke et al. (2014)	Quantitative – cohort	Gender‐ and age‐matched controls (total AN group only)	Total AN = 32	—	White = 31 (96.88%, mixed white/black caribbean = 1 (3.22%)	18	AN	Traits – AQ	Inpatient services	Treatment completion, treatment duration, ED symptoms
Li et al. (2020)	Quantitative – clinical audit	Low autistic traits	Total AN = 476 IP: HAT = 86 (28.1%) SU: HAT = 20 (24.4%) DC: HAT = 20 (22.7%)	IP: 85 (98.8%) SU: 19 (95%) DC: 20 (100%)	IP: white = 271 (88.6%), black = 2 (0.7%), mixed = 5 (1.6%), asian = 14 (4.6%), other ethnic background = 14 (4.6%) SU: white = 66 (80.4%), black = 3 (3.7%), mixed = 4 (4.9%), asian = 3 (3.7%), Other ethnic background = 6 (7.3%) DC: white = 80 (91%), mixed = 4 (4.5%), asian = 3 (3.4%), Other ethnic background = 1 (1.1%)	18+	AN	Traits – AQ‐10	Inpatient and day treatment	Clinical outcomes (BMI, ED symptoms, depression/anxiety, work/social functioning, motivation for change)
Nazar et al. (2018)	Quantitative – RCT	AN only	Total AN = 149 Autistic traits = 23 Possible/probable autism diagnosis = 6	Autistic traits = 95.65% possible/probable autism diagnosis = 100%	Caucasian = 143 (96%)	13–21	AN	Traits – DAWBA	Inpatient and day treatment	Type of treatment, frequency of service use, ED symptoms, BMI, psychosocial outcomes
Parsons (2023)	Quantitative – retrospective records review	EDs only	Total = 40 historical diagnosis = 4 new diagnosis = 5 clinical suspicion = 11	100%	White = 29 (72.5%), hispanic = 10 (25%), asian = 1 (2.5%)	13–25	EDs	Traits – medical notes	Partial PHP level of care	Treatment duration, treatment completion (discharge, graduation, completion)
Stewart et al. (2017)	Quantitative – clinical audit	Low autistic traits	Total = 286 (high ASD traits = 20/6.9%)	20 (100%)	NR	> 18	AN	Traits – DAWBA, SAS, AQ	Inpatient	Treatment duration, physical health, ED symptoms
Susanin et al. (2022)	Quantitative – case series RCT	Low autistic traits	Total AN = 59	—	White = 55 (93.2%)	12–18	AN	Traits – AQ‐10	Inpatient (CRT or FBT)	Treatment completion, weight gain, BMI
Diagnosis										
Babb et al. (2021)	Qualitative (inductive, essentialist thematic analysis) – Interviews	—	15	15 (100%)	NR	18+	AN	Diagnosis (self‐confirmed, screened using AQ‐10)	Community	Experiences of ED services
Babb et al. (2021)	Quantitative – survey	REDs only	REDs only = 110 autism + REDS = 46	46 (100%)		18+	RED	Diagnosis (service‐ or self‐confirmed)	Community (experience of ED treatments and/or services)	Type of treatments/services, treatment duration, treatment experiences (ratings)
Bentz et al, (2022)	Quantitative case series	REDs only	REDs only = 141 autism + REDS = 16		—	> 18	RED	Diagnosis (ADOS, ADI)	Inpatient (FBT)	Intensified care, weight normalization, treatment duration, successful ending of treatment
Pruccoli et al. (2021)	Quantitative – case series	Low autistic traits	Total AN = 22	20 (90.9%)	NR	11–17	AN	Diagnosis (ADOS)	Community and ED services	Frequency of treatment, duration of treatment, ED symptoms, body weight, OCD, depression
Tchanturia et al. (2019)	Quantitative – clinical audit	No autism/not assessed for autism			NR	Adults (18+)	AN	Diagnosis (ADOS)	Inpatient services	Treatment duration
Zhang et al. (2022)	Quantitative – cohort	Non‐Autistic AN	Total AN = 3055 (Autism = 134)	AN = 98% autism = 95%	NR	16+	AN	Diagnosis (ICD codes, two occurrences of diagnosis)	ED treatment overall, inpatient, day patient	ED symptom severity and persistence, ED treatment
Mixed										
Kinnaird et al. (2019)	Qualitative (codebook thematic analysis) – semi‐structured interviews	—	13	11 (84.6%)	NR	18+	AN	Mixed – traits (AQ‐10, *n* = 4) diagnosis (ADOS, *n* = 9)	Community and ED treatments	Experiences of ED treatments
Tchanturia, Larsson, and Adamson (2016)	Quantitative – case series	Low Autistic traits	Total AN = 35 (high ASD = 14)		NR	Adults (18+)	AN	Mixed – traits (AQ‐10, *n* = 8), diagnosis (ADOS, *n* = 27)	Inpatient (CRT)	Cognitive outcomes, motivation for change

Abbreviations: ADI = Autism Diagnostic Interview; ADOS = Autism Diagnostic Observation Schedule; AQ‐10 = Autism Quotient (10‐item); AQ = Autism Quotient; DAWBA = Development and Wellbeing Assessment; ICD = International Classification of Diseases; SCQ = Social Communication Questionnaire; SRS = Social Responsiveness Scale.

^**a**^ It was the authors intention to report a level of education/socio‐economic status column for review study characteristics; however, no included study reported these statistics.

### 3.2 Critical Appraisal

All studies were assessed to have clear research aims and purposes, as well as whether the data collected suitably addressed the research questions. The remaining studies were assessed against independent criteria for across three Quantitative methods (Randomized Controlled Trials (RCTs;*n* = 3), non‐RCTs (*n* = 10) and Descriptive (*n* = 2)) and one Qualitative category (n = 2).

Two of the studies assessed against Quantitative/RCT criteria (Nazar et al. 2018; Giombini et al. 2022) were rated to be of fair quality, with unclear information about groups, outcome data, and blinding of the researchers raising bias concerns. The final RCT study (Susanin et al, 2022) was rated as good quality. Several studies in the Non‐RCTs did not control for possible confounders in their study, either through matching groups in a suitable way or by controlling for key variables (e.g., age, sex, BMI) in their analyses. Additionally, there were concerns over the representativeness of samples, with raters unable to tell if participants were representative of the broader Autistic population from around half of the studies. Overall, however, studies were rated to be of good quality, generally using appropriate measures and consistent administration of the extended intervention or exposure (Stewart et al. 2017; Huke et al. 2014; Tchanturia et al. 2019; Adamson et al. 2018; Pruccoli et al. 2021; Li et al. 2022b; Babb et al. 2021; Bentz et al. 2022; Zhang et al. 2022), with the exception of Dandil et al. (2020), which was deemed as fair quality. Of the two studies assessed against the Quantitative/Descriptive criteria, Babb et al. 2022 was rated as very good quality and Tchanturia, Larsson, and Adamson (2016) as good quality. Both studies assessed against the Qualitative criteria (Kinnaird et al. 2019; Babb et al. 2021) were rated to be of very good quality. Please see Supporting Information (Tables S1–S4) for the full critical appraisal criteria and scoring for each included study.

### 3.3 Integrated Results Synthesis

Results will be presented for studies that had samples with an autism diagnosis (including two studies who used a mixture of diagnosis and traits, as both studies reported a majority diagnosed sample), followed by studies that used Autistic traits. ED treatment outcomes and experiences have been organized into *Eating disorder treatment utilization* (Types of ED services and treatments; Duration of ED treatment; Treatment completion rates), *Eating disorder‐specific outcomes and experiences* (ED symptoms, Body Mass Index) and *Broader eating disorder recovery outcomes and experiences* (Socio‐emotional outcomes, Cognitive outcomes, Co‐occurring psychopathology outcomes, Broader social outcomes). See Table 2 for a summary of study results.

**TABLE 2:** Key results of included studies (*n* = 17), organized into studies that used Autistic traits (*n* = 9), autism diagnosis (*n* = 6) or both (*n* = 2) in their sample.

Author	N=	ED	Outcomes	Controlled for	Results	Key findings
Traits						
Adamson et al. (2018)	CREST‐I = 66 (AN+ASD = 21) CREST‐G = 62 (AN+ASD = 21)	AN	Social anhedonia, alexithymia (socio‐emotional functioning) Self‐reported motivational ability (ability to change and importance to change)	BMI	CREST‐G: sig effect of time on ability to change in whole group (F(1,48) = 4.57, *p* = 0.04) CREST‐I: sig effect of time on alexithymia scores in whole group (F(1,47) = 6.84, *p* = 0.01) and ability to change (F(1,37) = 11.27, *p* > 0.01)	CREST in group format may improve self‐reported ability to change, while CREST in individual formats may improve alexithymia and self‐reported ability to change = CREST possibly a useful intervention for both groups
Dandil et al. (2020)	AN = 49 Autism + AN = 50	AN	Cognitive outcomes (central coherence, set shifting)		Small sig effect of autistic traits on central coherence scores (*F*(136) = 3.93, *p* < 0.05), and on cognitive rigidity (*F*(57) = 10.45, *p* < 0.01) All participants improved on set‐shifting (*t*(60) = 8.57, *p* < 0.001) and central coherence (*t*(58) = −2.35, *p* < 0.05), with no sig difference between those who scored above and below cut‐off Autistic trait scores for set‐shifting (*t*(97) = 0.02, *p* > 0.05) or central coherence (*t*(96) = 0.52, *p* > 0.05). Neither groups improved on central coherence (autism + AN = *t* (34) = −1.44, *p* > 0.05; AN only = *t*(23) = −1.9, *p* = 0.06)	
Giombini et al. (2022)	Total AN = 80 Autism and AN = 18	AN	Cognitive outcomes (set‐shifting, cognitive flexibility, central coherence) ED symptoms, depression, motivation, social communication, autistic traits, IQ and clinical outcomes (height, weight, duration of illness, lowest BMI, medication and no. of previous hospital admissions)	Cognitive outcomes (set‐shifting, cognitive flexibility, central coherence) ED symptoms, depression, motivation, social communication, Autistic traits, IQ and clinical outcomes (height, weight, duration of illness, lowest BMI, medication and no. of previous hospital admissions)	Treatment group, age, autism group Autism + AN group performed better in the Brixton test in the Delayed condition when CRT was delivered at a later stage of the treatment (*p* < 0.041, *η* ^2^ *p* = 0.054) AN only group performed better in the WCST in the immediate condition when CRT was delivered at the initial stage of the treatment (*p* < 0.037, *η* ^2^ *p* = 0.057). Those in the older young person group (AN only) responded positively (*p* = 0.040, *η* ^2^ *p* = 0.058), while younger YP and Autism + AN did not. Non‐sig effects of treatment groups on central coherence, cognitive rigidity or motiviationmotivation to change following treatment	Autism + AN group performed better in the Brixton test in the delayed condition when CRT was delivered at a later stage of the treatment (*p* < 0.041, *η* ^2^ *p* = 0.054) AN only group performed better in the WCST in the immediate condition when CRT was delivered at the initial stage of the treatment (*p* < 0.037, *η* ^2^ *p* = 0.057). Those in the older young person group (AN only) responded positively (*p* = 0.040, η^2^p = 0.058), while younger YP and autism + AN did not. Non‐sig effects of treatment groups on central coherence, cognitive rigidity or motiviation to change following treatment Delayed timing of CRT may be more beneficial when delivered later in treatment for Autistic people when looking to improve cognitive outcomes.
Huke et al. (2014)	AN = 32 Autism + AN not reported	AN	Treatment completion		No significant effect of Autistic traits on premature termination of treatment (*p* = 0.101)	Suggests no difference between Autistic and non‐Autistic groups on treatment completion
Li et al. (2020)	Total AN = 476 IP: HAT = 86 (28.1%) SU: HAT = 20 (24.4%) DC: HAT = 20 (22.7%)	AN	BMI, ED symptoms, depression/anxiety, work/social functioning, motivation for change		Inpatient care: higher Autistic traits predicted improvements in ED symptoms at end of treatment (B = 0.97, *p* = 0.048, Nagelkerke's *R* ^2^ = 19.6%) 30.3% of high Autistic traits no longer met BMI cut‐off for AN (< 17.5) at the end of treatment vs. 19.5% with low autistic traits (fishers exact *p* = 0.05) Those with high Autistic traits had higher on anxiety (*p* = 0.013, Hedge's *g* = 0.46), depression (*p* = 0.009, biserial correlation *r* = 0.22) and work and social functioning (*p* = 0.008, biserial correlation *r* = 0.22) outcomes after treatment. Day care: lower Autistic traits predicted improvement in ED symptoms at the end of treatment (*B* = −3.06, *p* = 0.047, Nagelkerke's *R* ^2^ = 44.3%) No sig group differences on BMI, ED symptoms, anxiety, depression, work/social functioning, motivation for change in day care or step‐up programs	No group differences across all settings on BMI/weight restoration, with Autistic group showing stronger improvements following inpatient care Those with high Autistic traits showed significant poorer psychological and work/social outcomes following the end of inpatient care
Nazar et al. (2018)	AN = 126 Autistic traits = 23 (Possible/probable autism diagnosis = 6)	AN	Type of treatment, frequency of service use, ED symptoms, BMI, strengths & difficulties (peer problems, prosocial difficulties, hyperactivity, emotional problems, conduct problems)		Those with Autistic traits had sig more inpatient and day‐patient days (*U* = 1077.0, *z* = 2.433, *p* = 0.015), more frequently admitted to an ED specialist inpatient treatment (18.2% vs. 5%); (*X* ^*2*^ = 6.62; *p* = 0.02) Both groups had similar increases in BMI and weight for height from baseline to 12 months, as well as similar reductions in ED symptoms Sig higher general difficulties reported at 12‐month FU in Autistic group (*U* = 49.0, *z* = −3.10, *p* = 0.002)	Suggests that autistic people with an ED have longer and more frequent inpatient treatment, as well as more significant psychosocial difficulties following treatment, while groups have similar BMI/weight outcomes
Parsons (2023)	Total = 40 Historical diagnosis = 4 New diagnosis = 5 Clinical suspicion = 11	ED	Treatment duration, treatment completion (discharge, graduation, completion)		Sig correlation between Autistic traits and number of calendar days in treatment (*r* = 0.388, *p* = 0.013), with 15.8% of variation in duration down to percentage of Autistic traits Non‐sig differences across discharge, graduation and completion on level of Autistic traits	Suggests those with high Autistic traits require longer treatment duration, while there is no impact of autistic traits on meeting requirements to graduate from treatment
Stewart et al. (2017)	Total AN = 286 Autism + AN = 20	AN	Treatment completion, weight gain, BMI	Depression, anxiety	Longer treatment duration reported in high Autistic trait groups (*χ*2 = 7.30, df = 1, *p* < 0.01), reflected in both admission to an intensive day patient program (*χ*2 = 6.21, df = 1, *p* < 0.025) and to psychiatric wards (*χ*2 = 8.68, df = 1, *p* < 0.005) but not in greater use of pediatric medical admission (*χ*2 = 0.27, df = 1, *p* > 0.05) Parent‐reported depression as sig predictor of longer treatment duration (*B* = 0.04, *p* < 0.01) No sig differences in weight gain and BMI between high and low Autistic traits groups	Total AN = 286 Autism + AN = 20
Susanin et al. (2022)	Total AN = 59	AN	Treatment completion, weight gain, BMI		No sig links between Autistic traits and treatment drop out (*χ* ^2^ (1) = 0.00, *p* = 0.98), treatment intensity (*χ* ^2^(1) = 0.70, *p* = 0.40), weight gain (*t*(26) = −0.37, *p* = 0.72, Hedge's *g* = −0.14) or BMI (*t*(35) = −0.56, *p* = 0.58, Hedge's *g* = −0.20)	No association between higher Autistic traits and ED treatment outcomes
Diagnosis						
Babb et al. (2022)'s	REDs only = 110 Autism + REDS = 46	REDs	Type of treatments/services, treatment duration, treatment experiences		Non sig differences in treatment duration (*t*(148) = 0.936, *p* > 0.05, 95% CI [−0.1, 0.28], Hedges' g_s_ = 0.17) Sig higher percentage of autistic + REDs group reported having accessed CAMHS (*X* ^2^ (1) = 9.65, *p* < 0.01). Autism + REDs group reported accessing significantly more care settings (*t*(148) = 2.704, *p* < 0.01, 95% CI [0.15, 0.93], Hedges' *g*_s_ = 0.49) and significantly more ED treatments (*t*(148) = 1.176, *p < 0*.05, 95% CI [−0.15, 0.58], Hedges' *g*_s_ = 0.44). Autism + REDs group rated ED service setting (*t*(136) = −2.062, *p < 0*.05, 95% CI [−1.05, −0.02], Hedges' *g*_s_ = 0.38), ED treatment received (*t* (146) = −2.53, *p < 0*.05, 95% CI [−1.12, −0.14], Hedges' *g*_s_ = 0.46) and psychological therapies received (*t*(116) = −2.261, *p < 0*.05, 95% CI [−1.33, −0.09], Hedges' *g*_s_ = 0.45) as sig less beneficial	No differences in treatment duration, but autism + REDs groups reported accessing more ED treatments and a poorer experience of ED services, treatments and therapies
Babb et al. 2021	15	AN	Experiences of ED services		Themes: (1) Misunderstanding autism – participants discussed multiple referrals due to misunderstanding or lack of interest from ED clinicians; participants discussed that a therapeutic focus on body, weight and shape was not felt to the as relevant to their ED (2) One treatment does not fit all – participants discussed negative experiences (e.g., group therapy) and positive experiences (e.g., emotion‐focused, dieticians, OTs) within treatment, as well as challenges associated with CBT (3) Improving accessibility and engagement within services – participants discussed the importance of adapting communication styles and adapting service environments	Suggests that autistic people have negative experiences of ED services, underpinned by feeling misunderstood and poorly supported by ED clinicians and current treatments
Bentz et al. (2022)	REDs only = 141 Autism + REDS = 16	REDs	Intensified care, weight normalization, successful end of treatment, duration of treatment		A significantly larger proportion of autism and REDs group (50%) received intensified care at some point during their treatment, compared with REDs only group (16%) No sig differences between weight normalization and successful ending of treatment No sig differences in treatment duration	Suggests Autistic people with REDs require a higher level of care than non‐Autistic with REDs No group differences in weight normalization, ending of treatment or treatment duration
Pruccoli et al. (2021)	AN = 17 Autism + AN = 5	AN	Frequency of treatment, duration of treatment, BMI, ED symptoms, weight, OCD, depression		Non‐sig group differences on treatment frequency or duration, BMI, ED symptoms, psychological outcomes, depression or OCD	Suggests that high Autistic traits are not associated with treatment intensity or outcomes
Tchanturia et al. (2019)	Not reported	AN	Inpatient admissions	Not applicable	Mean admission length for patients in 6‐year period before autism‐specific clinical pathway introduced higher in Autistic group (133 day/19 weeks) than non‐Autistic (109 days/16 weeks) Following implementation of clinical pathway, Autistic group showed lower mean length of admissions (90 days/13 weeks) then non‐Autistic (118 days/17 weeks)	Suggests that the introduction of a specialized clinical care pathway reduced treatment duration for Autistic people
Zhang et al (2022)	Total AN = 3055 (Autism = 134)	AN	ED treatment received	Gender, age	Treatment duration and intensity significant higher in Autistic group (vs. non‐Autistic group) Outpatient (ORs = 2.39, (95% CI 1.61–3.55)) Tube feeding (OR = 5.31 (95% CI 2.74–10.29)) Number of inpatient days with AN (122 days in Autistic group vs. 28 in non‐autistic, IRR = 4.49 (95% CI 2.54–7.94))	Suggests Autistic people with AN significantly more likely to use outpatient and inpatient services, as well as to receive tube feeding. Suggests Autistic people have sig longer inpatient treatment duration
Mixed						
Kinnaird et al. (2019)	13	AN	Experience of ED treatments		Themes: (1) Relationship between autism and anorexia – participants discussed how their Autistic traits impacted their anorexia in “non‐traditional” ways (e.g., more focus on control, sensory differences and social confusion as opposed to desire to lose weight or body image issues) (2) Problems with treatment – participants discussed difficulty accessing treatments in the first instance or being refused treatment, difficulties with traditional timeframes and how their social, sensory and communication differences impacted their engagement with treatment (3) Treatment adaptations – participants discussed emotion‐focused approaches, sensory adaptations, longer timeframes and focusing more on improving overall quality of life, as well as the importance of ED clinicians recognizing their autism	Suggests that Autistic people may have different ED presentations than non‐Autistic peers, as well as difficulties accessing and engaging with current ED services
Tchanturia, Larsson, and Adamson (2016)	Total AN = 35 (High ASD = 14)	AN	Cognitive outcomes (cognitive flexibility, attention to detail), motivation for/ability to change		Sig differences on the cognitive rigidity subscale (flexibility) in the low scoring ASD group (*p* > 0.01, *d* = 0.5) and self‐reported ability to change (p > 0.01, *d* = 0.5) after treatment No sig differences on any scales for high ASD group No sig group differences on attention to detail (F (1,33) = 3.32, *p* > 0.05) or motivation to change (F (1,32) = 1.47, *p* > 0.05) Sig differences reported on change in ability to change (F (1,32) = 6.59, *p* = 0.02), direction not reported	Suggests that CRT may be more effective for those with low Autistic traits

Abbreviations: ADI = Autism Diagnostic Interview; ADOS = Autism Diagnostic Observation Schedule; AQ‐10 = Autism Quotient (10‐item); AQ = Autism Quotient; DAWBA = Development and Wellbeing Assessment; ICD = International Classification of Diseases; SCQ = Social Communication Questionnaire; SRS = Social Responsiveness Scale.

### 3.4 Autism Diagnosis

#### 3.4.1 Eating Disorder Treatment Utilization

##### 3.4.1.1 Type of ED Services and Treatments

Zhang (2022)'s cohort study, rated to be of high quality, found that the Autistic individuals had three times higher odds of receiving inpatient care (46% compared to 23%). This study also reported a significant association between having received an autism diagnosis and use of different intensities of ED services, with the largest effect sizes reported for increased receipt of tube feeding (OR = 5.31; Zhang 2022). Bentz et al (2022) also found that a significantly larger percentage of Autism and REDs group (50%) received intensified care at some point during their treatment, compared with REDs only group (16%; *p* = 0.004).

In contrast, however, Babb et al (2022) did not find group differences between Autistic and non‐Autistic individuals with REDs on the different types of ED services used, types of treatment or psychological therapy received. However, there were significant group differences reported on their *experience* of these services; Autistic individuals with RED reported poorer experiences with ED treatment settings (*t*(136) = 2.062, *p* < 0.05, 95% CI [−1.05, −0.02], Hedges' *g*_s_ = 0.38) and ED treatments overall (*t*(116) = −2.261, *p < 0*.05, 95% CI [−1.33, −0.09], Hedges' *g*_s_ = 0.45), with medium effect sizes reported in both findings. Thus, Babb et al's (2022) study would suggest that both Autistic and non‐Autistic groups use the same range of ED services and treatments, however Autistic individuals have poorer experiences of ED treatments than their non‐Autistic peers.

Babb et al's (2022) finding regarding poorer experiences on ED services is supported and further contextualized by findings from an inductive, essentialist thematic analysis of interviews that reported participants with an autism diagnosis described negative and anxiety‐inducing experiences of psychological therapies (Babb et al. 2021). For example, in a group therapy context one participant reflected that a lot of Autistic people “go into shutdown in these groups, become silent, become withdrawn and find it hard to properly engage” (page 1414, Babb et al. 2021). Encouragingly, Autistic participants discussed aspects of ED treatment that they had more positive experiences with, such as emotion‐ and practical‐focused therapies such as Dialectical Behavior Therapy (DBT) or Occupational Therapy (OT) (Babb et al. 2021). In a similar vein, participants in Kinnaird et al. (2019)'s mixed sample study discussed difficulties accessing ED treatments in the first place, which is an important consideration when interpreting contrasting quantitative findings; if Autistic individuals are having difficulty accessing ED services, it could be that only a small sample of Autistic experiences are being represented in quantitative studies exploring treatment duration and utilization.

##### 3.4.1.2 Duration of ED Treatment

Zhang et al. (2022) found that an autism diagnosis was significantly associated with increased number of days spent in inpatient units (Autistic with AN = 122 days vs. non‐Autistic with AN = 28 days, IRR = 4.49). Tchanturia et al. (2019) reported service inpatient duration before and after the implementation of the PEACE pathway, a novel clinical care service for Autistic people with AN, with a reported change from 19 weeks for Autistic people and 16 weeks for non‐Autistic people to 13 weeks and 17 weeks respectively (Tchanturia et al. 2019). However, it should be noted that this latter study was a descriptive study and did not statistically compare groups on treatment duration.

Conversely, three studies did not find significant differences in treatment duration between Autistic and non‐Autistic individuals with ED (Babb et al. 2022; Bentz et al. 2022; Pruccoli et al. 2021) Additionally, all three studies looked at Autistic people with a RED, with Pruccoli et al. 2021 focusing on AN patients only. Importantly, two of these studies looked at treatment duration out with in‐patient care; Babb et al. 2022 looked at treatment duration within ED services generally (*t*(148) = 0.936, *p* > 0.05, 95% CI [−0.1, 0.28], Hedges' *g*_s_ = 0.17) while Bentz et al. (2022) looked at duration of FBT specifically. While it is hard to draw firm comparative conclusions give that studies focus different ED services and treatments, evidence from both Autistic traits and diagnosed autism samples tentatively suggest that prolonged treatment duration may be specific to inpatient services.

Findings from Babb et al. (2021)'s qualitative study provide insight into reported differences in treatment duration. with participants discussing multiple referrals during their engagement with ED services. This was often felt to be down to misunderstandings from ED clinicians about Autistic presentations of EDs, leading to participants feeling that clinicians just “didn't want to know” (page 1413, Table 3, Babb et al. 2021), or that there was a notable lack of understanding from neurotypical clinicians on Autistic communication styles and patterns (Babb et al. 2021). Further contextualizing quantitative study results, participants in Kinnaird et al. (2019)'s mixed sample study reported that participants described difficulty with typical timeframes of treatment, feeling that they needed longer time to process and engage with support than their non‐Autistic peers. Thus, it could be that reported differences in treatment duration, particularly within inpatient settings, may be explained by poor understandings and accommodations for Autistic characteristics within ED services.

##### 3.4.1.3 Treatment Completion Rates

One study looked at treatment completion rates in participants with an autism diagnosis reporting non‐significant results (Bentz et al. 2022). Thus, quantitative evidence from both Autistic traits and diagnosed autism samples suggests that there is no difference in treatment completion outcomes compared to lower Autistic traits or non‐Autistic peers. However, qualitative findings in Kinnaird et al. (2019)'s mixed sample study highlighted that misunderstandings between the Autistic person and the ED clinician often led to self‐ or clinician discharge from treatment: “The first facility I went to, before the autism diagnosis was problematic because they thought that I was stubborn and lazy and unwilling to help myself, and they let me know it. They ended up asking me not to come back, because my case was too “complex”” (Kinnaird et al. 2019, page 5,). This would suggest that a wider lens may be needed in studies exploring treatment completion rates, taking into account group differences or experiences in self‐ or formal discharge within services.

#### 3.4.2 Eating Disorder‐Specific Treatment Outcomes and Experiences

##### 3.4.2.1 ED Symptoms

One quantitative study looked at ED symptoms as a treatment outcome in those who had received an autism diagnosis, reporting non‐significant differences between Autistic and non‐Autistic participants (Pruccoli et al. 2021). Qualitative studies that explored ED symptoms may help to contextualize these findings. Interestingly, whilst it was not evident in differing efficacy from the quantitative findings, Autistic participants reported that traditional ED treatments felt less well suited to their specific presentations. Both Babb et al. (2021) and Kinnaird et al. (2019) reported that Autistic participants felt that a therapeutic focus towards food, weight, and body image were not the most relevant issues to the development of their ED and that less ‘traditional’ factors play a more important role in their ED. Indeed, some participants in Kinnaird et al. (2019)'s mixed‐sample study emphasized that ED recovery for Autistic people should be less associated with these behaviors and should be more focused on other factors, such as how much [is] that person is able to now be like engaging in life (page 313).

##### 3.4.2.2 Body Mass Index (BMI)

Studies with samples with an autism diagnosis reported non‐significant differences in weight normalization and BMI following FBT (Bentz et al. 2022) and general ED services (Pruccoli et al. 2021).

#### 3.4.3 Broader Eating Disorder Recovery Outcomes and Experiences

##### 3.4.3.1 Socio‐Emotional Outcomes

There were no quantitative studies that looked at socio‐emotional outcomes in samples with an autism diagnosis, however findings from qualitative studies provided insight into Autistic experiences of socio‐emotional outcomes. There was consensus amongst diagnosed Autistic participants that emotion‐focused therapies, such as DBT, are more successful due to helping Autistic people recognize, manage and regulate their emotions better: “DBT was really helpful…we spent a long, long time talking through emotions, identifying them and trying to think about how to regulate, how to manage them…DBT was about [emotion regulation] skills, and that was really important” (page 1413, Table 3, Babb et al. 2021). Similarly, participants in Kinnaird et al. (2019) study reported that social confusion and struggling to relate to others are important problems to consider during ED treatment, with participants suggesting that treatments may be more successful if they target socio‐emotional factors such as identifying and describing their emotions.

##### 3.4.3.2 Cognitive Outcomes

The complexity of cognitive factors in autism and EDs was highlighted by qualitative findings. Autistic participants in Babb et al. (2021) discussed struggling with abstract thinking and cognitive inflexibility required for interventions such as CBT. Other participants discussed how Autistic thinking styles and cognitions can in fact be harnessed during treatment, as some participants felt that more structure, routine, and rigidity around daily timetables in fact would make engagement with ED services easier, as opposed to running contrary to the standard neurotypical aim of ED treatments which focuses on reducing such factors.

Studies that used mixed samples further supported this complexity. Tchanturia, Larsson, and Adamson (2016) quantitative study found significant reductions in cognitive rigidity (*p* > 0.01, d = 0.5) and self‐reported ability to change (*p* > 0.01, d = 0.5) in the non‐Autistic group only, with no differences for those with higher Autistic traits, suggesting that treatment seeking to reduce cognitive rigidity in Autistic individuals and those with higher Autistic traits may be an ineffective treatment target. Qualitative findings shed further light on why interventions focusing on cognitive rigidity or adaptive ability may not work. For example, one participant in Kinnaird et al. (2019) study said “I think as somebody who's on the Autistic spectrum and who has an ED, I think AN is kind of like a product of who I am and how I think” (page 313), with other participants agreeing that they felt that the overlap between autism and ED traits was not felt to be accounted for in current ED treatment approaches or was poorly understood by ED clinicians.

##### 3.4.3.3 Co‐Occurring Psychopathology Outcomes

The only study which included a sample with a diagnosis of autism did not find significant differences between Autistic and non‐Autistic participants level of depression on discharge from treatment (Pruccoli et al. 2021), however it should be noted that a very small sample of Autistic people with AN (*n* = 5) was included in this analysis. However, findings from Kinnaird et al. (2019)'s qualitative provided further insight into Autistic experiences of co‐occurring psychopathology outcomes and mental health difficulties following ED treatment. Participants with both high Autistic traits and an autism diagnosis discussed how their mental health difficulties were poorly understood within ED services, and how their mental health difficulties—and their understanding of these difficulties—are keenly integrated with their autism: “Actually, all this mental health stuff that I've had—this is why, this is why I've felt like I've not fitted into the world for the last 45 years, and actually I'm just different and that's ok” (page 312, Kinnaird et al. 2019).

##### 3.4.3.4 Broader Social Outcomes

There were no studies included in the review that looked at broader social functioning outcomes in diagnosed Autistic samples.

### 3.5 Autistic Traits

#### 3.5.1 Eating Disorder Treatment Utilization

##### 3.5.1.1 Type of Ed Services and Treatments

Nazar et al. (2018) found that a higher percentage of AN participants with higher Autistic traits used ED specialist inpatient treatment when compared to those with lower Autistic traits (18.2% vs. 5.0%, *X* ^2^ = 6.62, *p* = 0.02). Similarly, Stewart et al. (2017) found that participants with higher Autistic traits (as measured by parent‐report AQ) had more frequent admissions to intensive day patient programs (*X* ^2^ = 6.21, df = 1, *p* < 0.025) and to psychiatric wards (*X* ^2^ = 8.68, df = 1, *p* < 0.005).

##### 3.5.1.2 Duration Of Treatment

Stewart et al. (2017), found that those with higher Autistic traits reported a longer duration of inpatient treatment compared to those with lower Autistic traits. This effect was also reported by Tchanturia, Larsson, and Adamson (2016) and by Nazar et al. (2018) who reported that those with higher Autistic traits had more general inpatient days (*U* = 1077.0, *z* = 2.433, *p* = 0.015). Finally, in a retrospective study of medical records, Parsons (2023) found a significant medium‐strength correlation between Autistic traits and number of calendar days in partial hospitalization treatments (*r* = 0.388, *p* = 0.013).

##### 3.5.1.3 Treatment Completion Rates

Three studies explored treatment completion rates in ED samples with higher Autistic traits, with all three studies reporting non‐significant differences compared to non‐Autistic groups (Huke et al. 2014; Susanin et al. 2022; Nazar et al. 2018).

#### 3.5.2 Eating Disorder‐Specific Treatment Outcomes and Experiences

##### 3.5.2.1 ED Symptoms

Only one study reported significant differences; at discharge from treatment, Stewart et al. (2017) found significant but small correlations between change in EDE‐Q scores and Autistic traits on all EDE‐Q subscales of weight concern (*r* = 0.290), shape concern (*r* = 0.269), and global score (*r* = 0.287; all *p* < 0.004), with significantly lower change associated with higher Autistic traits. The remaining studies did not report significant differences in ED symptoms following treatment between those with higher and lower Autistic traits, across inpatient and day patient services (Huke et al. 2013; Nazar et al. 2018), and specific ED treatments such as FBT (Susanin et al. 2022). Fletcher‐Watson (2022) found that low AQ‐10 scores predicted clinically significant improvements in ED symptoms in day patient care (*B* = −3.06, *p* = 0.047, Nagelkerke's *R* ^2^ = 44.3%), while having AQ‐10 scores predicted clinically significant improvements in inpatient care (*B* = 0.097, *p* = 0.0048, Nagelkerke's *R* ^2^ = 19.6%), although this approach does not allow one to draw conclusion about group differences.

##### 3.5.2.2 Body Mass Index (BMI)

Three studies reported non‐significant differences on BMI following ED treatment in those with higher Autistic traits compared to lower Autistic traits (Susanin et al. 2022 ; Fletcher‐Watson 2022; Stewart et al. 2017). Nazar et al. (2018) reported that those with both higher and lower Autistic traits demonstrated a similar increase in BMI and weight restoration, although it should be noted no analyses were run to statistically investigate group differences.

#### 3.5.3 Broader Eating Disorder Recovery Outcomes and Experiences

##### 3.5.3.1 Socio‐Emotional Outcomes

One quantitative study looked at the impact of group and individual emotional skills training intervention (Cognitive Remediation and Emotional Skills Training; CREST) in AN patients with high Autistic traits (Adamson et al. 2018). Authors reported heightened levels of alexithymia and social anhedonia in those with higher Autistic traits across both formats, and a significant reduction in alexithymia scores in the Group format at the end of treatment (Adamson et al. 2018). Importantly, there were no significant group differences in reduction of outcomes, suggesting that emotion‐focused interventions, particularly in an individual format, are effective for individuals with both higher and lower Autistic traits.

##### 3.5.3.2 Cognitive Outcomes

Dandil et al. (2020) reported that both high and low Autistic trait groups showed significant improvements set‐shifting (*t* (60) = 8.57, *p* < 0.001) and central coherence (*t*(58) = −2.35, *p* < 0.05) following individual CRT, with no significant differences reported between groups (*p* > 0.05). Interestingly, Fletcher‐Watson (2022) study similarly reported improvements in cognitive flexibility across groups with both high and low Autistic traits, but found that the timing of the intervention was important to efficacy. Specifically, AN participants who have high Autistic traits performed better on a set shifting task if CRT delivery was delayed until later in the treatment (*p* < 0.041, *η* ^2^ *p* = 0.054), while those with low Autistic traits performed better on a set shifting task if CRT was delivered at the start of treatment (*p* < 0.037, *η* ^2^ *p* = 0.057), reflecting medium effect sizes.

##### 3.5.3.3 Co‐Occurring Psychopathology Outcomes and Experiences

Fletcher‐Watson (2022) reported that, on admission to inpatient care, individuals with higher Autistic traits had significantly higher rates of anxiety (*p* < 0.001) and depression (*p* < 0.001) than those with lower Autistic traits, with a similar effect observed in step‐up programs for anxiety (*p* = 0.009) but not depression. This significant difference was retained during discharge from inpatient care for both anxiety (*p* = 0.013, Hedge's *g* = 0.46) and depression (*p* = 0.009, biserial correlation *r* = 0.22), with authors reporting that anxiety levels particularly were well above the threshold for clinically severe symptoms following discharge from inpatient care. Nazar et al. (2018) similarly reported elevated rates of mood problems in a higher Autistic trait subgroup only, as well as one patient with ongoing obsessive‐compulsive disorder (OCD). Furthermore, a significant reduction from baseline in mood problems was reported for lower Autistic trait participants (p < 0.001) but not for those with higher Autistic traits (*p* = 0.77), although there was a notable disparity in sample sizes (*n* = 91 compared to *n* = 19 respectively). Interestingly, there were no differences between those with higher and lower Autistic traits on anxiety and depression outcomes following day patient or step‐up programs in Li et al. (2020) study, tentatively suggesting that this may be uniquely associated with ED inpatient treatments.

##### 3.5.3.4 Broader Social Outcomes

Mirroring their findings on co‐occurring psychopathology outcomes, Li et al. (2020) reported that those with higher Autistic traits had significantly reduced occupational and social functioning (*p* = 0.008, biserial correlation *r* = 0.22) compared to those with lower Autistic traits following discharge from inpatient care, while levels of work and social functioning were well above the threshold for clinically severe concern. Authors reported that this effect was also in after step‐up programs with a medium effect size (*p* = 0.21, Hedge's *g* = 0.66). Similarly, Nazar et al. (2018) found evidence to suggest higher levels of general social, emotional and peer difficulties at follow up from inpatient and day patient care in AN participants with higher Autistic traits compared to lower Autistic traits (*U* = 49.0, *z* = −3.10, *p* = 0.002), however baseline scores were not reported.

## 4 Discussion

### 4.1 Summary of key findings

The current review adopted a mixed‐method approach to explore the impact of ED treatment on Autistic people and those with high Autistic traits. Emerging evidence suggests that Autistic people and those with higher Autistic traits report longer inpatient care, more frequent use of intensive treatment and poorer experiences across ED treatments than non‐Autistic peers. Similarly, both Autistic people and those with higher Autistic traits reported similar levels of ED symptoms and BMI following treatment compared to non‐Autistic peers or those with lower Autistic traits. Those with higher Autistic traits presented with more complex psychosocial difficulties that persisted following treatment, while Autistic individuals emphasized the significant impact of co‐occurring mental health difficulties. Emotion‐focused interventions may be effective, although the evidence base was small, and were felt to be more relevant than cognitive or behavioral support in Autistic individuals. The current evidence base is limited by an over‐reliance on Autistic traits as an indicator of autism, particularly with regard to broader eating disorder outcomes, and further research is needed in diagnosed Autistic samples in order to establish a solid evidence base that can inform ED treatments more suited to and positively experienced by Autistic individuals with an ED.

### 4.2 Eating Disorder Service Use

Findings from the review suggest that Autistic people and those with higher Autistic traits are at greater risk of inpatient admission, reporting multiple referrals and poorer experiences of ED treatment than their non‐Autistic peers or those with lower Autistic traits. Similarly, Autistic individuals and those with higher Autistic traits experienced longer ED treatment duration within inpatient services, possibly explained by difficulties with traditional treatment timeframes and a lack of understanding from ED clinicians. There could be several reasons why this may be the case. For example, it could be that they are more unwell on admission, given evidence to suggest that Autistic individuals with ED have been reported to present with more severe and enduring ED symptoms than their non‐Autistic counterparts (e.g., Leppanen et al. 2022). It could also be that current ED treatments and service ethos are not sensitive or tailored to autism‐specific characteristics or experiences of EDs. Encouragingly, one of the studies included in the review (Tchanturia et al. 2019) reported a reduction in treatment time following the implementation of a clinical care pathway modified specifically for Autistic individuals with AN. Building on this, we should continue to develop autism‐informed ED treatments and adapt existing treatments to meet Autistic characteristics and experiences. Collaboration between the autism and ED field should also be a priority, seeking to improve awareness and understanding of Autistic experiences of EDs within ED services.

### 4.3 Eating Disorder Symptoms

The current review did not found evidence for significant group differences in ED symptoms and BMI between Autistic and non‐Autistic individuals with EDs in both diagnosed and elevated Autistic traits samples. This is indeed a promising finding, however it is important to contextualize this within findings of more intensive, frequent and longer durations of care. Qualitative evidence can provide useful insights here, tentatively suggesting the potential for different underpinning mechanisms such as weight and shape driven cognition which were not always felt to be the most relevant to the Autistic experiences of EDs. This is contrary to leading ED theories (Fairburn, Cooper, and Shafran 2003) and measures (EDE‐Q; Fairburn and Beglin 1994) of EDs, such as anorexia nervosa (AN), builimia nervosa (BN) and binge eating disorder (BED), with the exception of Avoidant and Restrictive Feeding Intake Disorder (ARFID), a poorly understood ED that has been reported to notably overlap with autism (e.g., Koomar et al. 2021). The mixed‐method integration of evidence in the current review would therefore suggest that, while improvements in ED outcomes may be the same between groups, these improvements may be experienced differently in some Autistic individuals who report that food, weight, and body images disturbances are not as central to their experience of an ED. With regards to recovering from an ED, there is general consensus across Autistic and non‐Autistic individuals that recovery should place less emphasis on these factors (Kinnaird et al. 2019; Wetzler et al. 2020).

An important consideration here is the measure of ED symptoms used in the included studies, the majority of which was the EDE‐Q. Review findings suggest that current measures may therefore be based on neurotypical presentations of EDs and may not be as sensitive to autism‐specific presentations of EDs (Longhurst and Clark 2022). It is worth noting that none of the studies included in the review used an ED measure that has been validated in Autistic populations; indeed, to our knowledge, no ED measure to date has been previously validated in Autistic populations. Robust psychometric measures of Autistic presentations of EDs are therefore urgently needed before we can begin to draw firm conclusions regarding the severity of ED symptoms in Autistic individuals.

### 4.4 Cognitive, Socio‐Emotional and Psychosocial Outcomes

Several studies also looked at cognitive and socio‐emotional outcomes associated with specific ED interventions. Several studies looked at outcomes associated with Cognitive Remediation Therapy (CRT), an ED treatment that looked to address traits such as cognitive rigidity (Tchanturia, Larsson, and Adamson 2016; Dandil et al. 2020; Giombini et al. 2022), reporting mixed evidence for those with higher Autistic traits. Qualitative studies highlighted important concerns for such interventions, suggesting that factors such as cognitive rigidity or inflexibility are inherently associated with their autism (Kinnaird et al. 2019; Babb et al. 2021). Participants in Babb et al. (2021) study discussed how, in some instances, incorporating these factors into treatment might improve treatment (Babb et al. 2021), although this may not be the case for all. Thus, it is important to find the balance between accommodating for such factors in Autistic people or those with higher Autistic traits, and supporting those whereby cognitive rigidity has worked towards developing or maintaining disordered eating. This highlights the need for better understandings of ED‐ and autism‐specific factors and raises concerns of the potential harm of targeting Autistic traits or characteristics within ED treatments. Instead, emotion‐focused interventions may be a promising avenue for future studies (Kinnaird et al. 2019; Babb et al. 2021), in line with broader literature highlighting the importance of emotions in the overlap between autism and EDs (Beygui and Cascio, 2022).

There was general consensus across studies that co‐occurring psychopathology outcomes, particularly anxiety, and social and occupational outcomes are poorer in those with higher Autistic traits to compared to those with lower Autistic traits, with higher rates of anxiety and depression reported (Li et al. 2020; Nazar et al. 2018; Pruccoli et al. 2021). While the majority of quantitative studies looked at sample with higher Autistic traits, the impact of these mental health difficulties within ED treatment were highlighted by qualitative studies which drew on perspectives from those with an autism diagnosis (Kinnaird et al. 2019; Babb et al. 2021). Mental health difficulties, including the elevated rates of anxiety, depression and OCD reported and discussed by studies in the current review, are significantly elevated in Autistic populations (Hossain et al. 2020; Matson and Goldin 2013), with a detrimental impact on quality of life reported across development (Moss et al. 2017; Adams, Clark, and Keen 2019; Knüppel et al. 2018). Given that elevated rates of mental health difficulties were reported at both admission and discharge in those with high Autistic traits (e.g., Li et al. 2020), it is likely that these co‐occurring mental health difficulties may impact their experiences of their ED and their treatment. ED clinicians and healthcare professionals may benefit from increased awareness and understanding of the prevalence and burden of mental health difficulties in those with high Autistic traits, and future research should be conducted in samples of those with an autism diagnosis to further explore and understand this relationship.

### 4.5 The Mixed‐Methods Approach and Other Review Strengths

Collectively, the quantitative and qualitative studies included in the review helped shed novel insights onto the current state of the literature. The inclusion of qualitative evidence was integral in enhancing our understanding of longer inpatient stays and more frequent use of intensified care by discussing difficulties with traditional timeframes and more complex ED presentations, as well as identifying outcomes that could be targeted in future quantitative research (e.g., emotion‐focused therapies and outcomes). It also identified some discrepancies between similar levels of ED outcomes reported by the quantitative studies and poorer experiences reported by qualitative studies. Thus, the mixed‐method approach was a notable strength when approaching the research question, providing novel insights into how measurable ED treatment outcomes can be contextualized within Autistic experiences. It also highlighted the urgent need for clarification between reported outcomes and experiences when it comes to Autistic presentations of EDs. Another notable strength of the review was the multi‐dimensional consideration of treatment outcomes and experiences, considering not just weight or ED symptoms but also the psychosocial, emotional, and cognitive factors that are integral to the Autistic experience.

### 4.6 Limitations and Directions for Future Research

The current review is not without limitations. Across studies, there was a reliance on the use of Autistic traits, warranting caution when interpreting studies. Importantly, while this may be a valid interim indicator of autism (Westwood et al. 2016), it should not be taken as a confirmation of a diagnosis of autism. Self‐report measures exploring Autistic traits, such as the AQ‐10, are commonly used due to the time‐consuming nature of a robust autism assessment process; however, they are designed to be a screening rather than diagnostic tool. Future research is needed to address this current limitation of the evidence base and to empirically validate Autistic experiences of an ED reported in qualitative studies and within the autism and ED community. Critically, self‐report measures such as the AQ‐10 fail to account for the developmental requirements associated with an autism diagnosis. Collecting this developmental information is one way that future researchers can recruit more representative samples, as well as providing a more nuanced understanding of the complex interplay between Autistic traits, eating behaviors, and disordered eating. As an interim measure to more time‐consuming clinical measures (e.g., ADOS), researchers could consider using shorter measures from the broader autism literature, such as a 5‐item questionnaire that has been used to assess childhood and adult socio‐communicative differences in Autistic adults (Stewart et al. 2021). Research should also recruit diagnosed samples, considering both formal and self‐diagnoses. This is aligned with a community‐led shift towards self‐identification and positioning autism as an identity, while also serving to temporarily alleviate the impact from extensive neurodevelopmental assessment waiting lists within clinical services.

There was also an over‐reliance on quantitative over qualitative studies exploring ED outcomes in Autistic people or those with higher Autistic traits. Future research could use qualitative methodologies such as Interpretative Phenomenological Analysis (IPA; Smith, Flowers, and Larkin 2009) with the aim of understanding and interpreting the meaning of complex lived/living experiences (Tuffour 2017). Future research could also collect mixed‐method data, using lived/living experience perspectives to contextualize and improve measurable outcomes in real time. For example, Field et al. (2023) conducted a Delphi study that made clear and actionable recommendations for treatment adaptations, service organization and staff training, rooted in Autistic and stakeholder ratings and experiences. Finally, sensory processing differences, such as modality‐specific sensitivities (taste, smell, touch) and interoception (internal body sensations such as temperature, hunger and fullness) have been consistently implicated in the overlap between autism and EDs (Kinnaird, Stewart, and Tchanturia 2018; Kinnaird et al. 2020; Brede et al. 2020; Nimbley et al. 2022), and yet no quantitative study to date as explored the impact of ED treatment on sensory outcomes. It is important that sensory differences and experiences are explored in the context of ED treatment and recovery for Autistic people.

There are a lack of RCTs exploring ED interventions for Autistic people or those with elevated Autistic traits was also observed in the literature. Future research should work towards implementing RCT's for adapted or novel interventions drawing on or developed within autism‐informed frameworks. Careful and inclusive recruitment strategies will need to be adopted to recruit Autistic participants for these studies. While a full discussion of suggestions and approaches are out with the scope of this paper, the authors suggest increasing interdisciplinary research, knowledge exchange between the traditionally segregated autism and ED fields, and collaboration between health boards and services to recruit from multiple sites. Authors also suggest including elements of co‐production or participatory research design in these interventions, ensuring that interventions for Autistic people are guided by Autistic people and the priorities of the Autistic community.

Studies were also biased towards restrictive eating disorders (REDs), with 15 of the 17 included studies looked at ED treatment outcomes and experiences in AN sample and the other two studies in REDs. Thus, there was a notable lack of investigation into other feeding and eating diagnoses such as BED, BN, and ARFID, despite elevated Autistic traits being reported in all (Gesi et al. 2020; Carpita et al. 2022; Inoue et al. 2021). The overlap between autism or Autistic traits and non‐restrictive EDs are significantly under researched and therefore poorly understood. Finally, there was a stark lack of diversity reported across samples and no included study reported demographics regarding socio‐economic status. Despite the stereotype that EDs present primarily in affluent individuals, there is clear evidence of EDs in low‐income populations (Huryk, Drury, and Loeb 2021), possibly through associated factors such as food insecurity (Lydecker and Grilo 2019; Rasmusson et al. 2019) and cost of care (Ali et al. 2020).

## 5 Conclusions

Given the heightened prevalence of Autistic traits in ED populations, it is important that ED treatment outcomes and experiences of these populations are better understood. Drawing on both quantitative and qualitative research to ensure outcomes are contextualized within lived/living experience perspectives. The current review found that Autistic individuals and those with high Autistic traits report frequently using a broad range of ED services and treatments, particularly inpatient and more intensive care, as well as poorer experiences of ED services. Those with higher Autistic traits tend to present with more complex psychosocial difficulties that persist following ED treatment, with Autistic perspectives emphasizing that co‐occurring mental health and social difficulties should be carefully considered during treatment. Similar improvements in ED symptoms reported in quantitative literature were partially contradicted by qualitative reports of poorer outcomes and experiences, highlighting the importance of more robust, mixed‐method future studies exploring these research questions. Currently, the literature is limited by an over‐reliance on Autistic traits as an indicator of autism and more research is urgently needed to further elucidate trait versus state differences and to empirically validate Autistic experiences of EDs. Future research with diagnosed Autistic samples are also needed to understand ED treatment outcomes and to inform treatments that are more positively experienced. There also remains an urgent need for novel autism‐informed measures of ED treatment outcomes and experiences in Autistic samples, moving beyond the use of measures and interventions developed for neurotypical populations to those based on in‐depth understandings of Autistic characteristics and presentations of an ED.

## Author Contributions

**Emy Nimbley:** conceptualization, data curation, formal analysis, investigation, methodology, writing – original draft. **Helen Sharpe:** methodology, writing – review and editing. **Ellen Maloney:** writing – review and editing. **Karri Gillespie‐Smith:** writing – review and editing. **Kate Tchanturia:** writing – review and editing. **Fiona Duffy:** data curation, formal analysis, investigation, writing – review and editing.

## Conflicts of Interest

The authors declare no conflicts of interest.

## Supporting information


Data S1:

## Data Availability

Data sharing is not applicable to this article as no new data were created or analyzed in this study.
