# Supplementary material for: A Mixed Method Systematic Review Into the Impact of ED Treatment in Autistic People and Those With High Autistic Traits
Source: Int J Eat Disord. 2024 Nov 14;58(1):117–38. doi: 10.1002/eat.24311 (PMC11784838; doi:10.1002/eat.24311)
Supplement: Supplementary file 1 — Data S1: [file EAT-58-117-s001.docx]

Supplementary Material A – References and abstracts of non-English papers excluded from review

**Snouckaert, V. C., & Spek, A. A. (2020). Het ontstaan van anorexia nervosa bij mensen met een autismespectrum-stoornis; een kwalitatief en retrospectief onderzoek [The development of anorexia nervosa in people with an autism spectrum disorder; a qualitative, retrospective study]. *Tijdschrift voor psychiatrie, 62*(9), 760–767.**

To improve treatment options for women with autism spectrum disorder (ASD) in combination with anorexia nervosa (an), it is necessary to better understand which risk factors are involved in the development of an in this target audience. AIM: To identify risk factors for the development of an in people with ASD.METHOD: Six women with ASD who are, or have been suffering from an, were interviewed about the development of their eating disorder. The data were analyzed using the thematic analysis method. RESULTS: Regarding these participants, the generally accepted risk factors for developing an also appear to play a role, where it is possible that people with ASD form a vulnerable group for these risk factors. Characteristics specific for ASD were found to also contribute to the development of an in these participants. CONCLUSION: For women with ASD it is important to be aware of a vulnerability to develop an. Extra support in areas where they experience difficulties is therefore necessary. The ASD specific characteristics can be aggravating or sustaining factors for an and should therefore be included in the treatment.

**Spek A. A. (2015). Eetproblemen bij mensen met een autismespectrumstoornis zonder intellectuele beperking [Eating problems in individuals with autism spectrum disorder (ASD) but no intellectual impairment]. *Tijdschrift voor psychiatrie*, *57*(10), 749–756.**

**Background:**Little is known about the co-occurrence--in individuals--of autism spectrum disorder (ASD) and eating disorders and eating problems. Consequently, clinicians do not have enough information about how to diagnose or treat the combination of ASD and eating problems.

**Aim:**To discuss the scientific literature and clinical experiences relating to eating disorders and eating problems in people who have ASD but no intellectual impairment.

**Method:**The scientific literature was searched by means of PubMed, Medline and PsycINFO, and clinical experiences were discussed.

**Results:**The combination of ASS and anorexia nervosa seems to be a strong predictor that the eating disorder will follow a chronic course. It is not clear how often bulimia nervosa occurs in persons with ASD. Eating problems in persons with ASD often seem to be related to sensory sensitivity, eating preferences and motor problems. So far, little is known about the treatment of eating disorders and eating problems in individuals with ASD.

**Conclusion:**When diagnosing and treating eating disorders and eating problems in individuals with ASD, it is important to take information processing and behaviour characteristics of ASD into account. Further research is needed, particularly in order to cast more light on treatment possibilities.

Table S1 – Quantitative/RCTs studies (n=3)

| Author | Methodological quality criteria |  |  |  |  |
| --- | --- | --- | --- | --- | --- |
|  | Is randomization appropriately performed? | Are the groups comparable at baseline? | Are there complete outcome data? | Are outcome assessors blinded to intervention? | Did the participants adhere to the designed intervention? |
| Nazar et al (2018) | Y | Y | CT | CT | Y |
| Giombini et al (2022) | Y | CT | Y | CT | Y |
| Susanin et al (2022) | Y | Y | Y | CT | Y |

*Y = yes, CT = can’t tell*

Table S2 – Quantitative/non-RCTs studies (n=10)

| Author | Methodological quality criteria |  |  |  |  |
| --- | --- | --- | --- | --- | --- |
|  | Are participants representative of target population? | Are measurements appropriate regarding both outcome and intervention? | Are there complete outcome data? | Are confounders accounted for in design and analysis? | During the study period, is the intervention administered (or exposure occurred) as intended? |
| Adamson et al (2018) | CT | Y | Y | Y | Y |
| Babb et al (2022) | Y | Y | Y | Y | Y |
| Bentz et al (2021) | Y | Y | Y | N | Y |
| Dandil et al (2020) | CT | Y | N | N | Y |
| Huke et al (2013) | CT | Y | Y | CT | Y |
| Li et al (2020) | Y | Y | N | Y | Y |
| Parsons (2023) | CT | CT | Y | N | Y |
| Pruccolli et al (2021) | Y | Y | Y | N | Y |
| Stewart et al (2017) | CT | Y | Y | N | Y |
| Tchanturia et al (2016) | CT | Y | Y | Y | Y |
| Zhang et al (2022) | Y | Y | Y | Y | Y |

*Y = yes, N = no, CT = can’t tell*

Table S3 – Quantitative/Descriptive studies (n=2)

| Author | Methodological quality criteria |  |  |  |  |
| --- | --- | --- | --- | --- | --- |
|  | Is the sampling strategy relevant to address the research question? | Is the sample representative of the target population? | Are the measurements appropriate? | Is the risk of non-response bias low? | Is the statistical analysis appropriate to answer the research question? |
| Tchanturia et al (2020) | Y | CT | Y | N | CT |

*Y = yes, N = no, CT = can’t tell*

Table S4 – Qualitative studies (n=2)

| Author | Methodological quality criteria |  |  |  |  |
| --- | --- | --- | --- | --- | --- |
|  | Is the qualitative approach appropriate to answer the research question? | Are the qualitative data collection methods adequate to address the research question? | Are the findings adequately derived from the data? | Is the interpretation of results sufficiently substantiated by data? | Is there coherence between qualitative data sources, collection, analysis and interpretation? |
| Kinnaird et al (2019) | Y | Y | Y | Y | Y |
| Babb et al (2021) | Y | Y | Y | Y | Y |

*Y = yes*
